# A Case of Intraocular Pressure Elevation Secondary to Pupillary Occlusion

**DOI:** 10.7759/cureus.84917

**Published:** 2025-05-27

**Authors:** Masaki Tanito, Sho Ichioka, Chisako Ida, Hinako Ohtani, Keigo Takagi, Yuto Yoshida, Aika Tsutsui

**Affiliations:** 1 Department of Ophthalmology, Shimane University Faculty of Medicine, Izumo, JPN

**Keywords:** acute glaucoma, pupillary occlusion, pupillary seclusion, ultrasound biomicroscopy, yag laser membranotomy

## Abstract

We report a case of acute intraocular pressure (IOP) elevation caused by pupillary occlusion in a Japanese man in his 90s. The patient presented with a left frontal headache and vomiting, and ophthalmologic evaluation revealed marked IOP elevation, a shallow anterior chamber, and pupillary membrane formation obscuring the lens. While anterior segment optical coherence tomography (AS-OCT) was unable to confirm the lens position, ultrasound biomicroscopy (UBM) clearly demonstrated the absence of lens displacement or intraocular mass. Yttrium aluminum garnet (YAG) laser membranotomy promptly deepened the anterior chamber and lowered IOP, followed by successful cataract surgery. Differentiation from pupillary seclusion, another cause of secondary angle closure, is critical, as it requires distinct therapeutic approaches. This case highlights the diagnostic and therapeutic considerations in managing rare causes of acute glaucoma and serves as an instructive example for ophthalmology trainees.

## Introduction

Acute glaucoma is characterized by a sudden elevation of intraocular pressure (IOP) accompanied by visual loss, ocular pain, and headache [1]. The most common causes of acute glaucoma are primary angle-closure disease (PACD) due to relative pupillary block or plateau iris; however, in rare cases, angle closure from other mechanisms or elevated IOP with open angles may also be responsible [1]. In cases of acute glaucoma, it is crucial to promptly identify the underlying cause and initiate appropriate treatment. However, management can be challenging, particularly for less experienced physicians who often encounter such cases in emergency settings.

In addition to PACD, other mechanisms of angle closure that can cause acute glaucoma include pupillary block from lens or intraocular lens (IOL) displacement, aqueous misdirection, suprachoroidal hemorrhage, absolute pupillary block due to posterior synechiae (i.e., pupillary seclusion or iris bombe), and pupillary occlusion caused by a fibrous membrane [2,3]. Acute glaucoma caused by these distinct mechanisms requires different appropriate treatments; therefore, accurate diagnosis is crucial for prompt IOP reduction and the preservation of visual function.

Here, we report a case of acute glaucoma due to pupillary occlusion that was initially treated with yttrium aluminum garnet (YAG) laser membranotomy. This case holds significant educational value for ophthalmology residents and early-career clinicians, particularly in distinguishing angle closure from pupillary seclusion, as further discussed in the Discussion section.

## Case presentation

A Japanese man in his 90s presented to his local physician with a left frontal headache upon awakening and vomiting after breakfast. He was referred to the emergency department of Shimane University Hospital for brain imaging. Head CT revealed no abnormalities that could explain his symptoms. However, conjunctival injection in the left eye was noted, and he was referred to the ophthalmology department the same day.

His medical history included hypertension and hypothyroidism. He had undergone small-incision cataract surgery in the right eye three months earlier. The patient stated that he had not had useful vision in his left eye for many years and had not pursued surgery. He had no history of ocular trauma, intraocular inflammation, or other ophthalmic treatments besides the right eye surgery.

**Figure 1:**
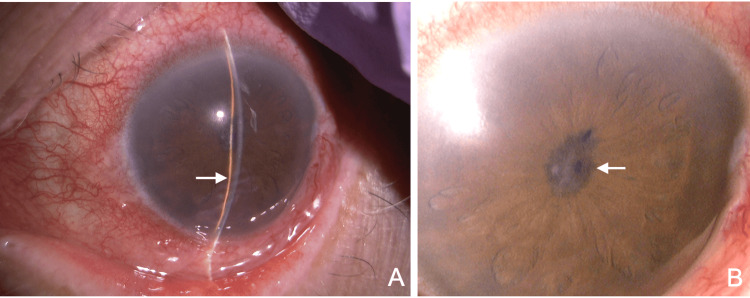
Slit-lamp findings during an acute glaucoma attack. (A) Marked shallow anterior chamber (arrow) and conjunctival injection are observed. Unlike PACD, mydriasis is absent. (B) The pupillary area is occluded by a fibrous membrane (arrow), and the lens is not visible. PACD: primary angle-closure disease

Ophthalmic examination by a resident revealed an IOP of 11.9 mmHg in the right eye and 48.6 mmHg in the left eye, measured with an iCare tonometer (Icare Finland Oy, Vantaa, Finland). The left eye showed conjunctival injection, corneal edema, a markedly shallow anterior chamber (Figure 1A), and pupillary occlusion (Figure 1B). No iris neovascularization was observed. Further evaluation by a senior ophthalmologist was performed to determine the treatment plan.

**Figure 2:**
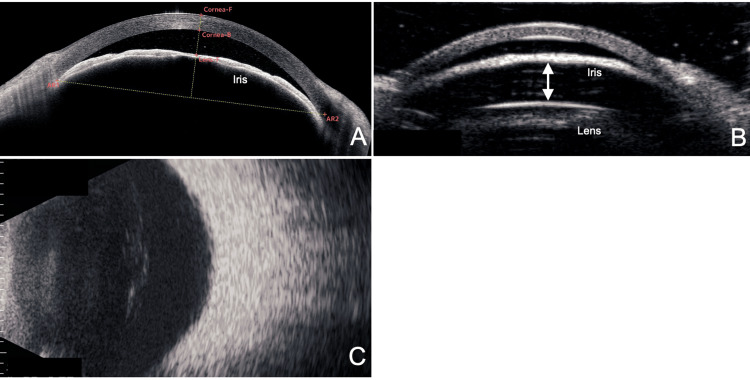
Anterior segment imaging using AS-OCT (A), UBM (B), and B-mode ultrasound (C). (A) A horizontal cross-section on AS-OCT shows anterior bowing of the peripheral iris, but the relationship with the lens remains unclear. (B) A horizontal UBM section reveals a space between the iris and lens (double arrows). (C) B-mode ultrasound shows no evidence of hemorrhage or mass lesion in the posterior segment. F, front; B, back; AR, angle recess; AS-OCT, anterior segment optical coherence tomography; UBM, ultrasound biomicroscopy

Anterior segment optical coherence tomography (AS-OCT; CASIA 2, Tomey Corporation, Nagoya, Japan) revealed anterior bowing of the iris in continuity with the pupillary membrane (Figure 2A). Because the lens position could not be identified on AS-OCT, ultrasound biomicroscopy (UBM; UD-8000, Tomey Corporation, Nagoya, Japan) was performed, showing a clear space between the iris and the lens, ruling out lens displacement (Figure 2B). B-mode ultrasound (UD-800, Tomey Corporation) revealed no evidence of intraocular hemorrhage or space-occupying lesions (Figure 2C). Blood tests did not indicate any systemic inflammatory disease. The patient was diagnosed with acute IOP elevation secondary to idiopathic pupillary occlusion.

On the same day, YAG laser membranotomy was performed using a YC-200 S Plus laser (Nidek Co., Ltd., Gamagori, Japan) at a power of 2.0 mJ with five shots. The anterior chamber deepened immediately after the procedure. To prevent reclosure due to inflammation, a peripheral laser iridotomy was also performed at the 2 o’clock position (3.0 mJ, 15 shots). Topical 0.1% betamethasone and a combination of 0.5% timolol/1% dorzolamide were prescribed twice daily.

**Figure 3:**
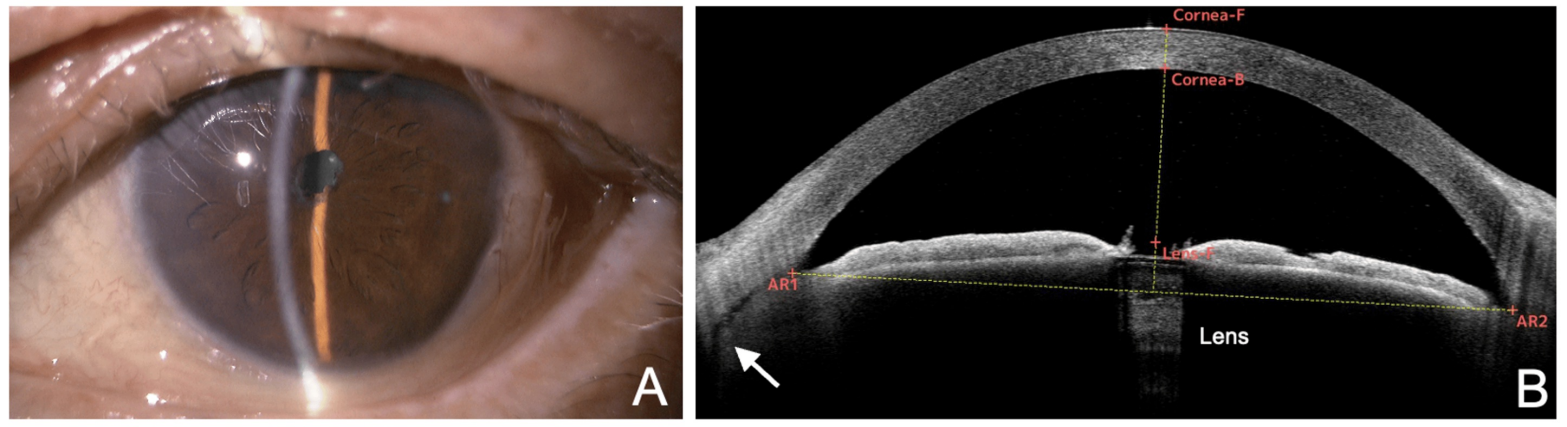
Slit-lamp (A) and AS-OCT (B) findings five days after membranectomy. (A) Increased anterior chamber depth is noted, and conjunctival injection has resolved. (B) Horizontal AS-OCT section reveals deepening of the anterior chamber and clear visualization of the lens. Mild ciliary body detachment is observed (arrow). F, front; B, back; AR, angle recess; AS-OCT, anterior segment optical coherence tomography

At the follow-up visit five days later, best-corrected visual acuity (BCVA) was 1.2 in the right eye and 0.03 in the left. IOP measured with a Goldmann applanation tonometer was 13 mmHg in the right eye and 10 mmHg in the left. The cornea of the left eye had cleared, the anterior chamber was deep, and no inflammatory signs were observed. Both the membranotomy and iridotomy sites remained open (Figure 3A). AS-OCT revealed mild ciliary body detachment, so cataract surgery was deferred until recovery (Figure 3B). At this time, optical biometry (OA-2000, Tomey Corporation, Nagoya, Japan) was possible, revealing axial lengths of 22.38 mm in the right eye and 22.05 mm in the left. The topical 0.5% timolol/1% dorzolamide was discontinued.

Four weeks after the IOP spike, cataract surgery was performed (Video 1). Anesthesia was induced with topical oxybuprocaine and intracameral 0.5% lidocaine. Pupil dilation was achieved with a double-hook technique. Phacoemulsification was performed through a 2.2 mm corneal incision, and a one-piece intraocular lens (+23.5 D, XY-1EM, HOYA Corporation, Tokyo, Japan) was implanted in the capsular bag. At the end of the surgery, 1 mg of betamethasone phosphate was injected subconjunctivally. Postoperatively, 1.5% levofloxacin and nepafenac were prescribed three times daily for two weeks.

**Video 1:** Intraoperative findings during cataract surgery.

**Figure 4:**
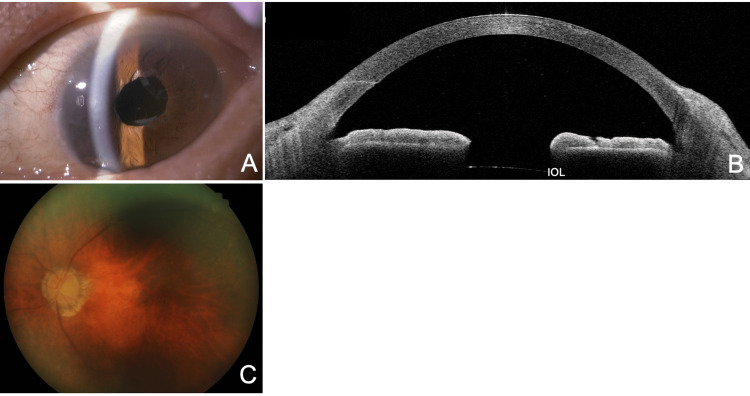
Observations two weeks after cataract surgery (six weeks after the initial IOP attack). (A) An IOL is implanted within the lens capsule. (B) The anterior chamber is further deepened. (C) No evident glaucomatous changes are seen in the optic disc. IOP, intraocular pressure; IOL, intraocular lens

Two weeks after cataract surgery, the BCVA in the left eye improved to 0.9 with -1.0 D correction, and IOP was 17.2 mmHg (iCare). The anterior chamber remained deep, with no inflammation, and the IOL was well-positioned (Figure 4A). AS-OCT showed a flattened iris and the resolution of ciliary body detachment (Figure 4B). Fundus examination revealed a vertical cup-to-disc ratio of 0.5 with no clear glaucomatous changes. Corneal endothelial cell density (EM-3000, Tomey Corporation, Nagoya, Japan) was 2019 cells/mm² in the right eye and 2412 cells/mm² in the left. Central corneal thickness was 581 µm in the right eye and 611 µm in the left. Anterior chamber flare measured with FM-600α (Kowa Company, Ltd., Nagoya, Japan) was 45.5 pc/msec in the right eye and 31.7 pc/msec in the left. The patient was referred to his local ophthalmologist for follow-up. At two months postoperatively, follow-up examination at the referral clinic showed left BCVA of 1.0 and IOP of 15 mmHg (iCare).

## Discussion

We encountered a case of acute glaucoma due to pupillary occlusion. While pupillary occlusion typically occurs secondary to chronic inflammation or trauma, the etiology in this case remained unknown. The patient’s advanced age made it difficult to determine the onset of the condition, which further complicated the search for a definitive cause. Elevated anterior chamber flare was also noted in the contralateral (right) eye, and although the exact timing of onset remained unclear, the pupillary membrane in the affected eye had recently progressed to complete occlusion. These findings suggested the possible presence of a systemic inflammatory condition as an underlying factor. However, as the patient declined further investigation, no additional systemic workup was performed.

Pupillary seclusion due to 360-degree posterior synechiae is an important differential diagnosis in cases of acute glaucoma secondary to chronic inflammation [2,3]. This distinction is critical because the treatment strategies differ: pupillary occlusion is best managed with YAG laser membranotomy, whereas pupillary seclusion typically requires synechiolysis or peripheral laser iridotomy/surgical iridectomy [4].

**Figure 5:**
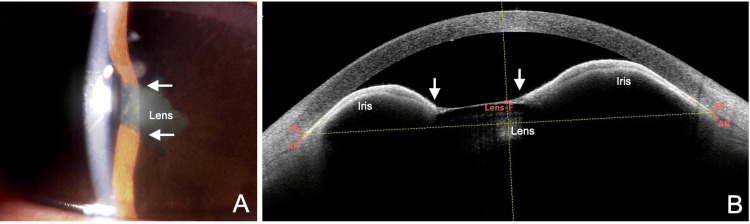
Slit-lamp (A) and AS-OCT (B) findings in a different case of seclusio pupillae secondary to uveitis. A woman in her 40s with right eye acute anterior uveitis and IOP of 56 mmHg (this case is also an original case included specifically for this manuscript). (A) 360-degree posterior synechiae of the iris (arrow) are noted. Unlike occlusio pupillae, the lens is visible in the pupillary area. (B) In seclusio pupillae, AS-OCT shows posterior synechiae (arrow) and archlike anterior bowing of the iris originating from both the sites of posterior synechiae and the angle recess (AR). The findings differ from those of occlusio pupillae. F, front; B, back; IOP, intraocular pressure; AS-OCT, anterior segment optical coherence tomography

A hallmark of pupillary occlusion is that the lens or intraocular lens (IOL) cannot be visualized through the pupil with slit-lamp examination due to the fibrous pupillary membrane. Since AS-OCT often cannot visualize posterior to the pupillary membrane, additional imaging with ultrasound biomicroscopy (UBM) or B-mode ultrasonography is necessary to rule out anterior lens displacement or intraocular space-occupying lesions. In contrast, in pupillary seclusion, the lens or IOL can usually be visualized through the pupil (Figure 5A). In rare cases, pupillary seclusion may also present with fibrin membranes covering the pupil, obscuring the lens; however, characteristic biphasic anterior bowing of the iris seen on AS-OCT or UBM is highly useful in distinguishing pupillary seclusion (Figure 5B).

In this case, initial treatment with YAG laser membranotomy, followed by cataract surgery, resulted in effective IOP control and significant visual improvement. Uveal effusion is frequently observed in the eyes with angle closure, particularly after IOP reduction [5-7]. In the present case, cataract surgery was performed after allowing time for the uveal effusion to resolve. Although pupillary occlusion and seclusion may occasionally be encountered in tertiary care centers, they are relatively uncommon in general practice. Moreover, current glaucoma treatment guidelines do not provide clearly defined criteria for these conditions [1]. This case is therefore considered to have substantial educational value for understanding the accurate diagnosis and appropriate management of acute glaucoma.

## Conclusions

This case emphasizes the necessity of thorough diagnostic evaluation and tailored therapeutic strategies in the management of uncommon etiologies of acute glaucoma, thereby providing meaningful educational value for ophthalmology trainees and clinical practitioners alike.
